# Vasorelaxant Effect of *Prunus mume* (Siebold) Siebold & Zucc. Branch through the Endothelium-Dependent Pathway

**DOI:** 10.3390/molecules24183340

**Published:** 2019-09-13

**Authors:** Cheolmin Jo, Bumjung Kim, Somin Lee, Inhye Ham, Kyungjin Lee, Ho-Young Choi

**Affiliations:** 1 Department of Herbal Pharmacology, Graduate School, Kyung Hee University, Seoul 02447, Korea; chocm456@naver.com; 2 Department of Herbal Pharmacology, College of Korean Medicine, Kyung Hee University, Seoul 02447, Korea; ori-pharm@hanmail.net (B.K.); iham@khu.ac.kr (I.H.); niceday@khu.ac.kr (K.L.); 3 Department of Biomedical Science and Technology, Graduate School, Kyung Hee University, Seoul 02447, Korea; sominleee@naver.com

**Keywords:** *Prunus mume* (Siebold) Siebold & Zucc., vasorelaxation, endothelium-dependent, hypertension, cardiovascular disease, Korean plum

## Abstract

Korean plum (*Prunus mume* (Siebold) Siebold & Zucc.) has long been used as a health food or herbal medicine in Asia. Previous studies have shown that several plants of the genus *Prunus* have vasodilatory and antihypertensive effects; we hypothesized that *P. mume* branches may have a vasorelaxant effect. In this study, we evaluated the effects and action mechanism of 70% ethanol extract of *P. mume* branch (PMB) on isolated rat aortic rings. Inhibitors such as NG-nitro-l-arginine methyl ester, 1*H*-[1,2,4]oxadiazolo[4,3-a]quinoxalin-1-one, methylene blue, indomethacin, atropine, tetraethylammonium chloride, glibenclamide, 4-aminopyridine and BaCl_2_ were used to investigate the mechanism of vasodilation responsible for the vascular relaxation. PMB (2–30 μg/mL) induced vasorelaxation in the presence of vascular endothelium, and all inhibitors used in this study affected the degree of relaxation. These results suggest that the vasorelaxant effect of PMB is endothelium-dependent and affects the nitric oxide-cyclic guanosine monophosphate pathway, prostacyclin pathway, muscarinic receptor pathway, and potassium channels. Our study explains that PMB may be another approach to hypertension treatment to reduce the burden of cardiovascular disease.

## 1. Introduction

Cardiovascular disease is the leading cause of death worldwide compared to other diseases [1], with hypertension (high blood pressure) being a major contributor to this mortality [2]. To reduce this effect, many researchers have been working extensively on drug discovery to improve hypertension treatment, such as angiotensin-converting enzyme inhibitors, angiotensin receptor blockers, calcium channel blockers, renin inhibitors, sympathoplegic agents, thiazide diuretics, α-adrenergic blockers, β-adrenergic blockers, and vasodilators [3]. Despite the research and development of various treatment strategies, the number of adults with high blood pressure worldwide increased from 594 million in 1975 to 1.13 billion in 2015 [4]. Consequently, there is a growing need for more efficient and stable approaches for the prevention and treatment of hypertension. Among other causes, abnormalities in the regulation of vascular smooth muscle contraction cause a change in blood pressure, and an isolated increase in vascular smooth muscle is known to cause hypertension [5]. Therefore, the regulation of vascular smooth muscle using natural products may be an alternative to the treatment and prevention of hypertension.

Korean plum, *Prunus mume* (Siebold) Siebold & Zucc., is a deciduous tree of the family Rosaceae with over 3000 years of cultivation history. This species is cultivated in Korea, China, and Japan for use as landscape trees or fruit trees [6]. The fumigated fruit of *P. mume* has been used as the traditional medicine, “Omae”, in Korea [7,8]. In addition, various parts of *P. mume* (i.e., fruit, flower, leaf, branch, seed, and root) have been used in traditional Chinese medicine. Specifically, the branch is called “Meigeng” (in Chinese) and has been used to prevent abortion [8].

In the field of basic research, the *P. mume* flower has been demonstrated to be involved in antioxidant activity [9] and inhibitory effects against aldose reductase and platelet aggregation [10]. *P. mume* fruit inhibits the proliferation of cancer cells such as Hep-2, HEC-1-B, and SK-OV-3 [11], decreases adrenocorticotropic hormone levels in menopausal rat models caused by ether stress [12], improves the fluidity of human blood ex vivo [13], and inhibits growth signals of vascular smooth muscle cells induced by angiotensin II [14]. In addition, it exhibits antimicrobial [15] and hepatoprotective [16] activities. To date, there have been many studies on *P. mume* fruit and flowers, but little is known about the pharmacological effects of *P. mume* branch.

Previous studies have shown that *P. mume* fruit has no significant effect on blood pressure control in hypertensive patients [17], but various parts of the plants of genus *Prunus* such as *P. yedoensis* bark [18], *P. yedoensis* leaf [19], *P. seroina* leaf [20], *P. seroina* fruit [21], aerial parts of *P. lycioides* [22], and prunetin (a compound isolated from *P. yedoensis*) [23] have shown vasorelaxant and anti-hypertensive effects. Furthermore, studies on chemical compounds obtained from the branch of *P. mume* have led to the identification of flavonoids, triterpenoids, phenolic acids, and lactones [24]. Among these compounds, 3-*O*-caffeoylquinic acid (chlorogenic acid), caffeic acid, rutin, and luteolin are known to have vasorelaxant effects [25,26,27,28]. Thus, we hypothesized that *P. mume* branch might have vasorelaxant effects. In order to further validate the benefits of using *P. mume* branch in the fields of pharmaceutical and functional foods, this study investigated the vasorelaxant activities of the 70% ethanol extract of *P. mume* branch (PMB) and identified the mechanisms of the vasorelaxant activities.

## 2. Results

### 2.1. Vasorelaxant Effects of PMB in Rat Aortic Rings with Intact or Denuded Endothelium

**Figure 1:**
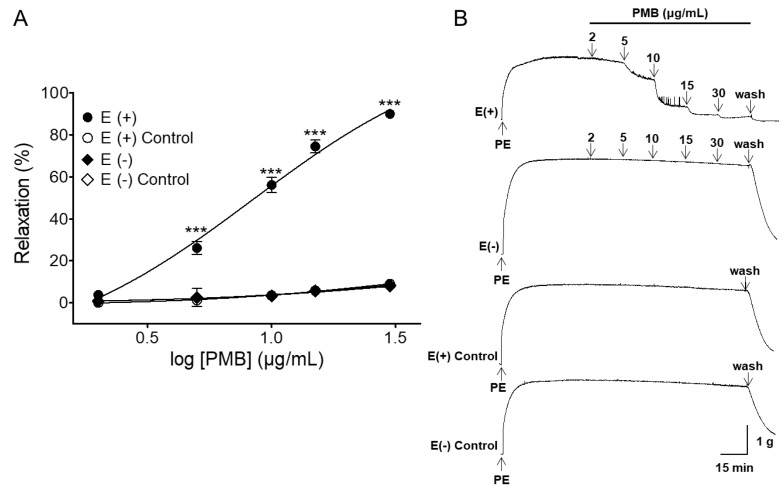
Cumulative concentration-response curves of PMB (2–30 μg/mL) in rat aortic rings with intact [(E+)] or denuded [(E−)] endothelium pre-contracted with phenylephrine (PE, 1 μM) (**A**). Representative traces under the indicated conditions (**B**). Values are expressed as mean ± SEM (*n* = 6). *** *p* < 0.001 vs. control.

PMB caused endothelium-dependent relaxation in endothelium-intact aortic rings pre-contracted by phenylephrine (PE, 1 μM) treatment. The maximal relaxation effect was 90.00 ± 0.73% (half maximal effective concentration (EC_50_) = 8.08 ± 1.03) and 7.83 ± 1.00% (EC_50_ = 11.74 ± 1.11) for endothelium-intact and endothelium-denuded aortic rings, respectively (Figure 1).

### 2.2. Vasorelaxant Effect of PMB on Endothelium-Intact Aortic Rings Pre-Incubated with NG-Nitro-l-Arginine Methyl Ester (l-NAME), Indomethacin, or Combination of l-NAME and Indomethacin

**Figure 2:**
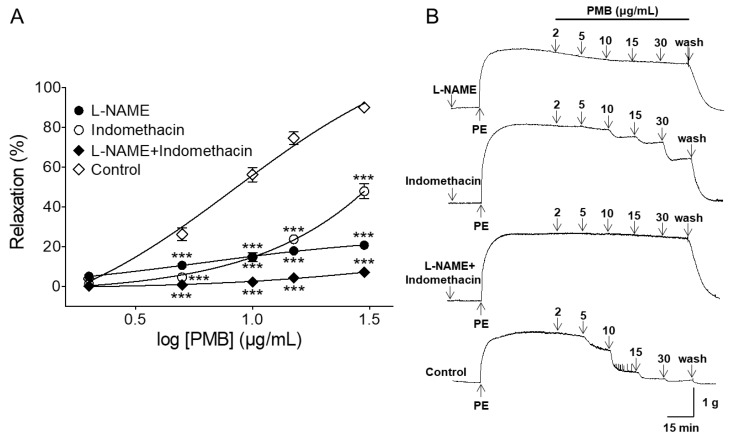
Concentration-dependent relaxation effect of PMB (2–30 μg/mL) on phenylephrine (PE, 1 μM)-pre-contracted endothelium-intact aortic rings in the presence and absence (control) of NG-nitro-l-arginine methyl ester (L-NAME, 100 μM), or indomethacin (10 μM), or a combination of L-NAME (100 μM) and indomethacin (10 μM) (**A**). Representative traces under the indicated conditions (**B**). Values are expressed as mean ± SEM (*n* = 6). *** *p* < 0.001 vs. control.

The PMB-induced vasorelaxant effect was significantly decreased by L-NAME (100 μM), an inhibitor of endothelial NO synthase (NOS). In the presence and absence of L-NAME, the maximal vasorelaxant effects were 20.66 ± 1.20% (EC_50_ = 7.31 ± 1.11) and 90.00 ± 0.73%, respectively. Pre-incubation with indomethacin, a cyclooxygenase inhibitor, significantly attenuated PMB-induced relaxation of endothelium-intact aortic rings pre-contracted by PE (1 μM). In the presence and absence of indomethacin (10 μM), the maximal relaxation effect was 47.83 ± 3.68% (EC_50_ = 14.18 ± 1.05) and 90.00 ± 0.73%, respectively. Pre-treatment with a combination of L-NAME (100 μM) and indomethacin (10 μM) significantly inhibited the vasorelaxant effect. The maximal relaxation effect was 7.00 ± 0.25% (EC_50_ = 12.40 ± 1.04) (Figure 2).

### 2.3. Vasorelaxant Effect of PMB on Endothelium-Intact Aortic Rings Pre-Incubated with 1H-[1,2,4]oxadiazolo[4,3-a]quinoxalin-1-One (ODQ) or Methylene Blue (MB)

**Figure 3:**
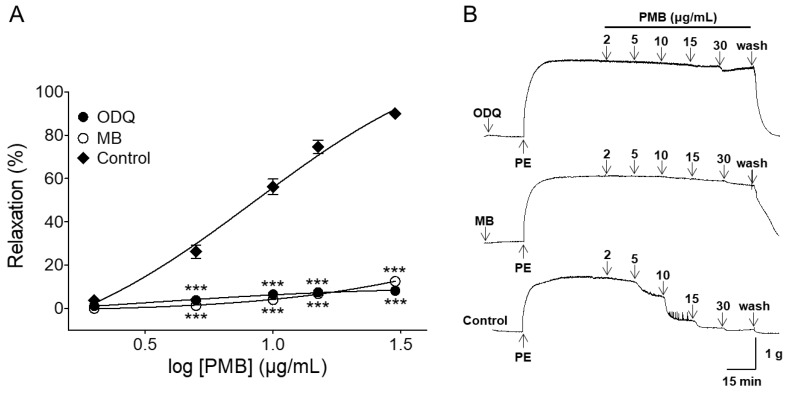
Concentration-dependent relaxation effect of PMB (2–30 μg/mL) on phenylephrine (PE, 1 μM)-pre-contracted endothelium-intact aortic rings in the presence and absence (control) of 1*H*-[1,2,4]oxadiazolo[4,3-α]-quinoxalin-1-one (ODQ, 10 μM) or methylene blue (MB, 10 μM) (**A**). Representative traces under the indicated conditions (**B**). Values are expressed as mean ± SEM (n = 6). *** *p* < 0.001 vs. control.

The PMB-induced vascular relaxant effect was significantly inhibited by the soluble guanylate cyclase (sGC) inhibitors 1*H*-[1,2,4]oxadiazolo[4,3-a]quinoxalin-1-one (ODQ, 10 μM) or methylene blue (MB, 10 μM). In the presence and absence of ODQ, the maximal vasorelaxant effect was 8.16 ± 0.40% (EC_50_ = 6.22 ± 1.10) and 90.0 ± 0.7%, respectively. In the presence and absence of MB, the maximal relaxant effect was 12.50 ± 0.84% (EC_50_ = 13.18 ± 1.04) and 90.0 ± 0.7%, respectively (Figure 3).

### 2.4. Vasorelaxant Effect of PMB on Endothelium-Intact Aortic Rings Pre-Incubated with Atropine

**Figure 4:**
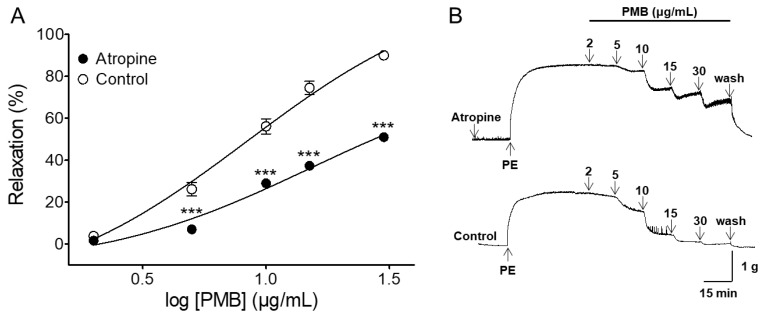
Relaxation responses induced by PMB (2–30 μg/mL) on phenylephrine (PE, 1 μM)-pre-contracted aortic rings in the presence or absence (control) of atropine (1 μM) (**A**). Representative traces under the indicated conditions (**B**). Values are expressed as mean ± SEM (*n* = 6). *** *p* < 0.001 vs. control.

Pretreatment of endothelium-intact aortic rings with atropine (muscarinic receptor antagonist) significantly diminished PMB-mediated vasorelaxation. In the presence and absence of atropine (1 μM), the maximal relaxation effect was 51.00 ± 1.50% (EC_50_ = 9.77 ± 1.03) and 90.0 ± 0.7%, respectively (Figure 4).

### 2.5. Vasorelaxant Effect of PMB on Endothelium-Intact Aortic Rings Pre-Incubated with Various Potassium Channel Blockers

**Figure 5:**
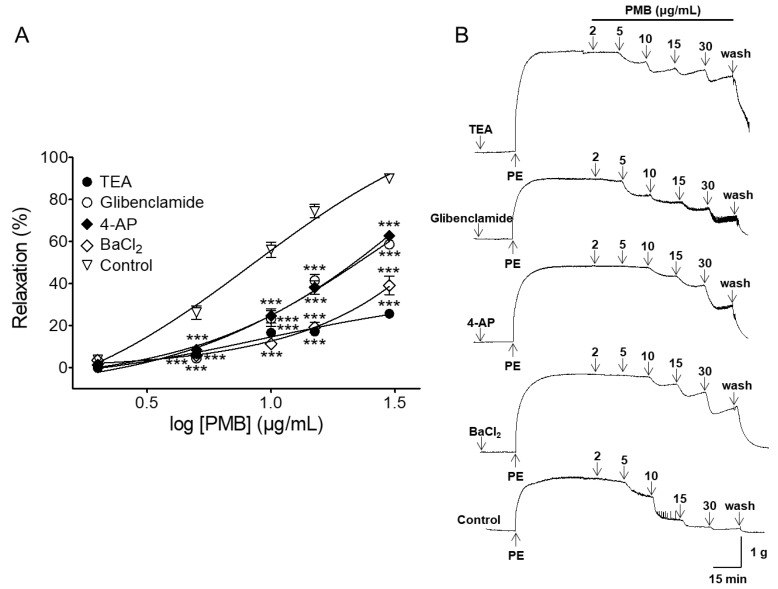
Relaxation responses induced by PMB (2–30 μg/mL) on endothelium-intact aortic rings pre-contracted with phenylephrine (PE, 1 μM) in the presence of tetraethylammonium (TEA, 5 mM), glibenclamide (10 μM), 4-aminopyridine (4-AP, 1 mM), or BaCl_2_ (30 μM) (**A**). Representative traces under the indicated conditions (**B**). Values are expressed as mean ± SEM (*n* = 6). *** *p* < 0.001 vs. control.

The PMB-induced vasorelaxant effect was significantly decreased by tetraethylammonium (TEA, 5 mM) and 4-aminopyridine (4-AP, 1 mM), the inhibitors of non-selective potassium channel. In the presence and absence of TEA or 4-AP, the maximal vasorelaxant effects were 25.66 ± 1.66% (EC_50_ = 8.39 ± 1.07) and 62.66 ± 1.89% (EC_50_ = 12.10 ± 1.04), respectively. Pre-incubation with glibenclamide (10 μM), an adenosine triphosphate (ATP)-sensitive potassium (K_ATP_) channel inhibitor, partially attenuated PMB-induced relaxation of endothelium-intact aortic rings pre-contracted by PE (1 μM). The maximal relaxation effect was 58.66 ± 1.97% (EC_50_ = 11.49 ± 1.03). Pre-treatment with BaCl_2_ (30 μM), the inward-rectifier potassium (K_IR_) channel blocker, significantly decreased the PMB-induced vasorelaxant effect. The maximal relaxation effect was 39.16 ± 4.38% (EC_50_ = 15.19 ± 1.06) (Figure 5).

### 2.6. Quantitative HPLC Analysis of Compounds in PMB

**Figure 6:**
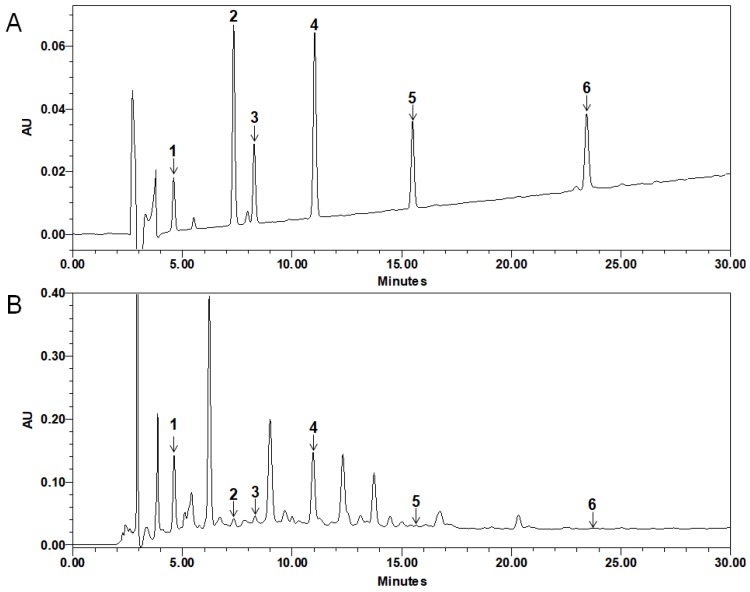
HPLC chromatogram of standard solution (**A**) and PMB (**B**) at 240 nm. 1; chlorogenic acid (retention time (RT) = 4.61 min), 2; caffeic acid (RT = 7.33 min), 3; rutin (RT = 8.30 min), 4; Genistein 7-*O*-β-glucopyranoside (G7G, RT = 10.96 min), 5; prunetin 5-*O*-β-glucopyranoside (P5G, RT = 15.66 min), 6; luteolin (RT = 23.65 min).

The retention time (RT) of chlorogenic acid, caffeic acid, rutin, genistein 7-*O*-β-glucopyranoside (G7G), prunetin 5-*O*-β-glucopyranoside (P5G) and luteolin were 4.61, 7.33, 8.30, 10.96, 15.66, and 23.65 min, respectively. Standard curve was calibrated by using the linear regression derived from the peak area. The regression equation (correlation coefficient, R^2^) of chlorogenic acid was y =11413x + 187,910 (0.9996), caffeic acid was y = 32,669x + 352,689 (0.9986), rutin was y = 12,975x + 86,657 (0.9999), G7G was y = 29625x + 118152 (0.9989), P5G was y = 13,269x + 78,669 (0.9999), and luteolin was y = 16,294x + 7079.5 (0.9999), which exhibited good linearity. The standard compounds content of PMB 1 g was as follows: chlorogenic acid was 133.01 ± 15.09, caffeic acid was 10.22 ± 10.55, rutin was 57.02 ± 4.50, G7G was 61.72 ± 3.73, P5G was 4.27 ± 5.58, and luteolin was 0.43 ± 0.09 μg (Figure 6).

## 3. Discussion

In the current study, PMB induced vasorelaxation in PE-pre-contracted endothelium-intact rat thoracic aorta. However, the effects of PMB on endothelium-denuded aortic rings were markedly reduced. These results suggested that the vasorelaxant effect caused by PMB was endothelium-dependent. Thus, we determined whether the mechanism of PMB vasorelaxation of the rat thoracic aorta was through an endothelium-dependent pathway and found that the vasorelaxant effect of PMB was related to NO-cGMP pathway, 4-aminopyridine prostacyclin (PGI_2_), muscarinic receptor pathway, and potassium channels. 

It is widely known that the vascular endothelium plays a vital role in vasorelaxation by secreting vasodilation substances such as NO, PGI_2_, and endothelium-derived hyperpolarizing factor (EDHF). In endothelial cells, an increase in calcium concentration induces calcium binding to calmodulin. The calcium-calmodulin complex stimulates NOS to activate NO formation from l-arginine. In smooth muscle cells, NO activates soluble guanylate cyclase and increases intracellular cGMP levels. As cGMP increases, calcium levels decrease and the smooth muscle relaxes [29,30]. In this study, L-NAME, ODQ, or MB were used to investigate the endothelium-depended vasorelaxation of PMB. The vasorelaxant effect of PMB was significantly attenuated by NOS inhibitor, L-NAME, and abolished by sGC inhibitors, ODQ and MB. Thus, the results obtained suggest that PMB induces a NO-cGMP pathway. 

PGI_2_, which is synthesized by cyclooxygenase, activates adenyl cyclase. Consequently, intracellular concentration of cyclic adenosine monophosphate increases, which leads to a reduction in the concentration of intracellular calcium in smooth muscles. As a result, the vascular smooth muscle relaxes [18,29]. In this study, indomethacin (cyclooxygenase inhibitor) significantly reduced the vasorelaxant effect of PMB. These findings indicate that PMB-induced vascular relaxation is mediated, in part, through NO- and COX-dependent pathways. In addition, the combination of L-NAME and indomethacin significantly inhibited the relaxation effect compared to single treatment with L-NAME or indomethacin. Thus, vascular relaxation of PMB may be exerted mainly through the NO-cGMP and PGI_2_ pathways.

Muscarinic receptors are present in vascular endothelial cells. Activation of muscarinic receptor on vascular endothelial cells causes increased synthesis of NO, which diffuses to adjacent vascular smooth muscle cells and causes vasodilation [31,32]. In this study, atropine, a muscarinic receptor antagonist, was shown to partially reduce the vasorelaxant effect of PMB. This result suggested that PMB regulates multiple pathways simultaneously, activating several receptors and channels, and might have a stronger tendency to activate other pathways as compared to the muscarinic receptors.

Activation of potassium channels both in the vascular smooth muscle and endothelium causes hyperpolarization which leads to vasorelaxation [33]. Apart from the endothelium-dependent and endothelium-independent vasodilatation mechanisms, potassium channels are a major mechanism involved in the regulation of vascular tone in vascular smooth muscle cells. To block potassium channels, we used the inhibitors TEA (5 mM) and 4-AP (1 mM), voltage-gated non-selective potassium channel blockers, glibenclamide (K_ATP_ channel blocker), and BaCl_2_ (K_IR_ channel blocker) [34,35]. *Prunus mume* branch-induced relaxation was partially decreased due to pretreatment with inhibitors, such as TEA, 4-AP, glibenclamide, and BaCl_2_. These data indicate the activation of potassium channels in the vascular smooth muscle and endothelium, including voltage-gated K_ATP_ or K_IR_ channels, may be involved in PMB-induced vasorelaxation.

Quantitative analysis was conducted on G7G and P5G, which are known to be present in the plant genus *Prunus* [36,37], and on chlorogenic acid, caffeic acid, rutin, and luteolin, which are reported to be present in *Prunus mume* branches [24]. The concentration of chlorogenic acid, caffeic acid, rutin, G7G, P5G, and luteolin in PMB was 133.01, 10.22, 57.02, 61.72, 4.27, and 0.43 μg, respectively. 

In previous studies, chlorogenic acid induced direct endothelium-dependent vasodilation by increasing NOS, cyclooxygenase, and EDHF pathways on rat aortic rings. The maximum effective concentration and the maximal effect values were 10^−4^ M and 67 ± 4%, respectively [25]. Rutin (10–160 μM) caused dose-dependent vasorelaxation by NO-cGMP pathway in endothelium-intact rings pre-contracted with PE. The maximal response value was 44.28 ± 7.48% [26]. Caffeic acid (10^–2^ g/L) caused relaxation activity against noradrenaline-induced contraction of rat aorta with/without endothelium [27]. Luteolin caused endothelium-dependent relaxation in endothelium-intact aortic rings pre-contracted by PE. Luteolin (4.5–36 μmol/L) caused a concentration-dependent relaxation in endothelium-intact or endothelium-denuded rat aortic rings precontracted with PE [28].

Quantitative analysis of PMB showed that the contents of chlorogenic acid and rutin, which cause endothelium-dependent vasorelaxation, were higher than those of other compounds. Therefore, the endothelium-dependent vasorelaxant effect of PMB may be related to compounds such as chlorogenic acid and rutin. However, since PMB possesses various known and unknown compounds, the vasorelaxant effect of PMB cannot be determined as the effect of only these two compounds. Thus, it is necessary to find more active compounds responsible for the vasorelaxant effects of PMB.

## 4. Materials and Methods

### 4.1. Chemicals and Reagents

Atropine, acetylcholine, CaCl_2_, caffeic acid, chlorogenic acid, ethylene glycol-bis(2-aminoethylether)-*N,N,N*′,*N*′-tetraacetic acid (EGTA), formic acid, glibenclamide, glucose, G7G, indomethacin, L-NAME, luteolin, MB, ODQ, PE, P5G, rutin, TEA, 4-AP were purchased from Sigma Aldrich, Inc. (St. Louis, MO, USA). KCl, KH_2_PO_4_, MgSO_4_ were purchased from Duksan Pure Chemicals Co., Ltd. (Ansan, Korea). BaCl_2_, NaCl, NaHCO_3_, and urethane were purchased from Daejung Chemicals & Metals Co., Ltd. (Siheung, Korea). Acetonitrile (HPLC grade) and Methanol (HPLC grade) were purchased from J.T. Baker Chemical Co., Ltd. (Phillipsburg, NJ, USA). ODQ, indomethacin, and glibenclamide were dissolved in dimethyl sulfoxide. Caffeic acid, chlorogenic acid, luteolin, P5G, G7G, and rutin were dissolved in methanol. All other drugs were dissolved in distilled water. 

### 4.2. Plant Material and Extraction

**Figure 7:**
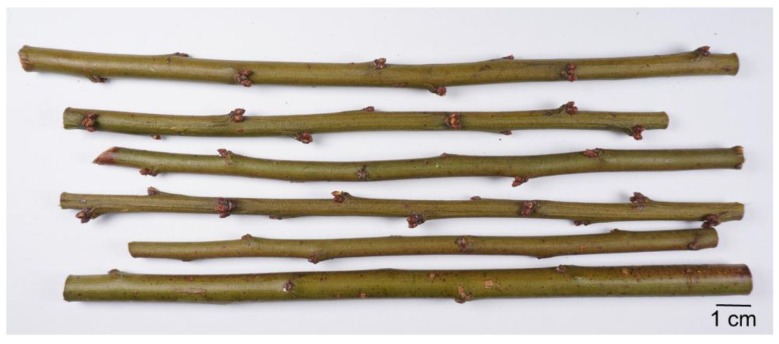
The branches (less than a year old) of *Prunus mume* (Siebold) Siebold & Zucc.

Branches of *P. mume* were collected from Jeongmi-myeon, Dangjin-si, Chungcheongnam-do, Republic of Korea in February 2018 (Figure 7). This plant was identified by Professor Kyungjin Lee of Kyung Hee University. A voucher specimen of the *P. mume* branch was deposited at the College of Korean Medicine, Kyung Hee University, Seoul, Republic of Korea. *P. mume* branches were washed in distilled water to remove contaminants. The plant material was chopped into small pieces and dried in a convection oven. Dried *P. mume* branches (280 g) were extracted two times with 70% ethanol for 2 h in a reflux apparatus. The extract was filtered with filter paper before being concentrated in a rotary evaporator under reduced pressure and subsequently freeze-dried. The yield of the extract was 13% (36.5 g). The 70% ethanol extract from the *P. mume* branch was completely dissolved in Krebs-Henseleit (KH) buffer.

### 4.3. Animals

Sprague-Dawley rats (SD, male, 220–260 g, 8 weeks-old) were purchased from Daehan Biolink (Chungbuk province, Korea). The animals were kept under a 12/12 h light/dark cycle and allowed free access to food and water. All procedures were executed according to the animal welfare guidelines and were approved by the Kyung Hee University Institutional Animal Care and Use Committee [KHUASP(SE)-18-074].

### 4.4. Preparation of Rat Aortic Rings

SD were anesthetized by urethane (1.2 g/kg, i.p.), and the thoracic aorta was removed and immersed in KH (composition (mM): NaCl, 118.0; KCl, 4.7; MgSO_4_, 1.2; KH_2_PO_4_, 1.2; CaCl_2_, 2.5; NaHCO_3_, 25.0; and glucose, 11.1; pH 7.4), maintained at 37 °C, and aerated with a mixture of 95% O_2_ and 5% CO_2_. After connective tissue and fat were carefully removed, approximately 2-mm-long aortic rings were cut and suspended in organ baths containing 10 mL KH at 37 °C. The rings in the organ baths were aerated with a mixture of 95% O_2_ and 5% CO_2_. The aortic rings were placed between 2 stainless steel wire hooks and connected to an isometric force transducer. After incubation under no tension for 20 min, the vessel segments were allowed to equilibrate for 40 min at a resting tension of 1.2 g. The KH was replaced every 20 min during the equilibration period. Changes in tension were recorded via isometric transducers connected to a data acquisition system (PowerLab, ADI instrument Co., New South Wales, Australia). The integrity of the endothelium was confirmed when acetylcholine (10 μM) caused >70% relaxation after pre-contraction by PE (1 μM). 

### 4.5. Experimental Protocols

Endothelium-intact and -denuded aortic rings were pre-contracted with PE (1 μM) in standard KH. After a 40 min equilibration period, cumulative concentrations of PMB (2, 5, 10, 15, and 30 μg/mL) were added to the organ baths. The relaxation effect of PMB on the aortic rings was calculated as a percentage of the contraction in response to PE.

To investigate the role of NO, PGI_2_, sGC, muscarinic receptors, or potassium channel in PMB-induced vasorelaxation, the following inhibitors were used: L-NAME (100 μM), an NOS inhibitor; indomethacin (10 μM), a cyclooxygenase inhibitor; combination of L-NAME (100 μM) and indomethacin (10 μM); ODQ and MB (10 μM), sGC inhibitors; atropine (1 μM), a muscarinic receptor antagonist; TEA (5 mM) and 4-AP (1 mM), non-selective potassium channel blockers; glibenclamide (10 μM), a K_ATP_ channel blocker; and BaCl_2_ (30 μM), a K_IR_ channel blocker. The rings were pre-incubated with each blocker for 20 min before contraction by PE. Each original register was checked, and the aortic rings used did not present significant elevation of the basal tone by pre-incubation with inhibitors, such as l-NAME (100 μM), indomethacin (10 μM), ODQ (10 μM), MB (10 μM), 4-AP (1 mM), TEA (5 mM), BaCl_2_ (30 μM), or glibenclamide (10 μM) in vascular contractile response to PE (1 μM).

### 4.6. Quantitative HPLC Analysis of Compounds in PMB

Precisely 100 mg of PMB was weighed and dissolved in 10 mL of methanol. The extract was then filtered through a 0.45 μm syringe filter. The standard compounds used for the quantitative analysis of PMB were chlorogenic acid, caffeic acid, rutin, G7G, P5G, and luteolin. One milligram of the standards was dissolved to give serial concentrations (50, 25, and 12.5 μg/mL for each sample) and HPLC chromatograms of the standards were obtained. The relationship between the concentration and the peak-area was measured by the minimum square method (R^2^ value). The HPLC apparatus was Alliance HPLC System equipped with a Waters e2695 separation module, a Waters 2998 Photodiode Array (PDA) detector (Waters Corp., Milford, MA, USA) and Empower 2 software (Waters Corp.). A YMC-Triart 4.60 × 250 mm C18 reversed-phase column with 5 μm particles was used. The mobile phase consisted of 0.1% formic acid (A) and acetonitrile (B) in a ratio specified by the following binary gradient with linear interpolation: 0 min 20% B, 30 min 40% B. The column eluent was monitored at UV 240 nm (PDA), following which all solvents were degassed with a micromembrane filter. Chromatography was performed at room temperature at a flow rate of 1 mL/min, and 10 μL was analyzed for 30 min. The quantity of the PMB was expressed as follows: the amount (mg) of standard compound = the quantitative amount (mg) of compound × AT/AS/n (n = 4; AT = the peak-area of the test sample containing the standard; AS = the peak-area of the standard).

### 4.7. Statistical Analysis

The values are expressed as the mean ± standard error of mean (SEM). Statistical analysis was performed using two-way ANOVA, and Bonferroni post-hoc test was conducted using GraphPad Prism 5 software (San Diego, CA, USA). The *p* < 0.05 was considered statistically significant. In all the experiments, n is equal to the number of aortic rings isolated from 2–3 rats. 

## 5. Conclusions

PMB caused endothelium-dependent vasorelaxation in rat aortic rings. The vasorelaxant activity of PMB were related to (1) NO-cGMP pathway, (2) PGI_2_ pathway, (3) muscarinic receptor pathway, and (4) potassium channels such as K_V_ channel, K_ATP_ channel, and K_IR_ channel. Further studies are needed to investigate the specific vasodilative compounds of PMB and the precise mechanisms of its action.
